# Therapeutic Thoughts

**Published:** 1911-05

**Authors:** James Burke

**Affiliations:** Grimms, Wis.


### THERAPEUTIC THOUGHTS.

By James Burke, M. D., Grimms, Wis.

If the use of cannabisis is followed by delirium it should be combined with veratrine, or with cicutine if movement is prominent. The hypnotic effect may be developed by adding a moderate dose of hyoscine or camphormonobromide.

For its anodyne effects the addition of menthol for the stomach, hyoscyanine for neuralgia, acetanilid for headache, should prove effective.

In fact, this is an admirable ingredient in the hands of the man who knows how to mix drugs to get their combined effects."

The interpretation of this learned paragraph is dubious as to whether the "author" intends to get the "physiologic action"—poisonous action of the principals used, or the biochemical neutralization of one by the other to assist the immunizizng faculty of the body in disposing of the accumulated proteid waste, as manifested by toxic symptoms, or both.

Prof. Robert Koch—the greatest bacteriologist—fell a victim to arterio-sclerosis. This malady is of toxic origin, the several causative toxins originating in bad physiology.

The immunizing faculty of his or any other person's body keeps the blood clear of the incidental surplus of riotous proteid waste, in strong people while yet young. But all faculties cease to be efficient beyond certain limits, and the riotous course of the proteid waste gets worse year by year. The study of physiology and the neutralizers of the toxins as they manifest themselves during life will obviate the too early onset of arterio-sclerosis.

Acidosis is one of the manifestations of proteid waste running riot in the blood, or being stored up in the body, conservatively.

The biochemic association of the lime and magnesia in the blood must be considered by every clinician in the treatment of disease.

The philosophy underlying the use of lime water emphasizes a long known clinical fact.

Many times a chronic disease or even many acute affections of the alimentary is greatly benefitted by this old remedy.

Magnesium infiltration of the tissues caused by the ingestion of salts, acidosis, or any other accident causing the physiologic lime content of the blood to deviate will lead to and produce nervous symptoms simulating insanity, paresis, bowel incapacity and many other "functional neuroses." The use of cicutine referred to in the quotation, evidently means the procurement of its poisonous effect on the periphal motor nervous tissue, excessive movement is thereby suppressed.

How does coiine (cicutine) do this? The bichemical arrangement of the tissue is temporarily changed.

Is there a disturbing toxin in the blood and in this tissue which excites the excessive movements which is biochemically neutralized by the cicutine? The removal of the toxic disturbance would always produce sedation—even in psychologic therapeutics, the removal of the contents of an impacted colon and rectum is sedative to the extent that their long presence has not established an abnormal affinity between them and the lining of the bowel to admit the absorption of morbific products into the blood.

There is a philosophy underlying our cold therapy that will be studied hard by the next generation.

Mixing drugs to get their combined effect sometimes is necessary in correcting the digestive enzymes and ferments, when iris, leptandra, chionanthus and prodo-phyllin, without catharsis, may be used. Hyoscyanine or any other drug is not universally useful in neuralgia. Often lime water will do better, especially if the physiologic association of lime and magnesium of the blood is disturbed. Clinical experience has taught us that the removal of the cause of the disturbance is a tonic, "hypnotic," "analgesic," and all the other good things we desire which we too often seek to secure by some standard treatment.

Eighty-five per cent. of "heart disease" is the result of toxicity of the blood from some curable cause.

